# The Action of Drugs in the Movements of the Stomach

**Published:** 1887-01

**Authors:** 


The Action of Drugs in the Movements of the Stomach.—Motor disturbances of the stomach play an important part in the diseases of this organ. Schütz has attempted to determine experimentally the effects of various drugs. The following are the conclusions:

1. *Excitants of the automatic centres*, with exaggeration of spontaneous movements, eructin, tartar emetic, apomorphia, and to a less extent strychnine, cafeine, veratrin, chloride of barium, nicotine and pilocarpine in small doses.

2. *Excitants of the nervous extremities* with general contraction of the stomach, muscarine.

3. *Increasing muscular excitability*, followed by tonic contraction of the stomach, phyrostigmine, digitaline, scillaine, helleborine.

4. *Paralyzers of the Automatic Centres.*—No substance totally destroys the movements of the stomach. Movements are diminished by chloral, urethan, morphine, pyrophosphates of zinc and arsenic, and by nicotine and pilocarpine in large doses.

5. *Paralyzers of the Nervous Extremities.*—Atropin; ether and chloroform abolish temporarily the excitability of the entire nervous apparatus of the stomach.—*Archix. exp. Pathologie.*

